# The effects of lumboperitoneal and ventriculoperitoneal shunts on the cranial and spinal cerebrospinal fluid volume in a patient with idiopathic intracranial hypertension

**DOI:** 10.3325/cmj.2016.57.293

**Published:** 2016-06

**Authors:** Ines Nikić, Milan Radoš, Ana Frobe, Miroslav Vukić, Darko Orešković, Marijan Klarica

**Affiliations:** 1 Croatian Institute for Brain Research, Zagreb University School of Medicine, Zagreb, Croatia; 2 Department of Oncology and Nuclear Medicine, Clinical Hospital Center “Sestre Milosrdnice,” Zagreb, Croatia; 3 Department of Neurosurgery, Clinical Hospital Center Zagreb, Zagreb, Croatia; 4 Department of Molecular Biology, Ruđer Bošković Institute, Zagreb, Croatia; 5 Department of Pharmacology, Zagreb University School of Medicine, Zagreb, Croatia

## Abstract

Lumboperitoneal (LP) and ventriculoperitoneal (VP) shunts are a frequent treatment modality for idiopathic intracranial hypertension (IIH). Although these shunts have been used for a long time, it is still not clear how they change the total craniospinal CSF volume and what portions of cranial and spinal CSF are affected. This report for the first time presents the results of a volumetric analysis of the total cranial and spinal CSF space in a patient with IIH. We performed an automated segmentation of the cranial and a manual segmentation of the spinal CSF space first with an LP shunt installed and again after the LP shunt was replaced by a VP shunt. When the LP shunt was in place, the total CSF volume was smaller than when the VP shunt was in place (222.4 cm^3^ vs 279.2 cm^3^). The difference was almost completely the result of the spinal CSF volume reduction (49.3 cm^3^ and 104.9 cm^3^ for LP and VP, respectively), while the cranial CSF volume was not considerably altered (173.2 cm^3^ and 174.2 cm^3^ for LP and VP, respectively). This report indicates that LP and VP shunts in IIH do not considerably change the cranial CSF volume, while the reduction of CSF volume after LP shunt placement affects almost exclusively the spinal part of the CSF system. Our results suggest that an analysis of both the cranial and the spinal part of the CSF space is necessary for therapeutic procedures planning and for an early recognition of numerous side effects that often arise after shunts placement in IIH patients.

Idiopathic intracranial hypertension (IIH) is a condition characterized by elevated intracranial pressure without evidence of structural intracranial abnormalities (1,2). Although IIH is usually diagnosed in obese adult females of childbearing age, it also affects the pediatric population, in which the sex distribution is more balanced (3). The typical symptoms of IIH are headache, nausea, vomiting, and visual impairment due to a development of papilledema. The treatment is primarily determined by the severity of headache and visual impairment, and ranges from conservative to surgical procedures (4). Unfortunately, surgical procedures (lumboperitoneal [LP] and ventriculoperitoneal [VP] shunting) are often associated with numerous complications, from shunt obstruction to over-drainage and tonsillar herniation (5-7). We report on a 6-year-old patient who developed the classic symptoms of IIH and was treated with conservative methods, as well as with LP and VP drainage. To the best of our knowledge, this is the first case report that describes changes in the cranial and spinal CSF volumes after LP and VP shunts placement. Our findings emphasize the crucial role of the spinal CSF space for compensation of various volume loads inside the CSF system.

## Case report

A previously healthy child was hospitalized at the age of 6 years due to headache, nausea, vomiting, and loss of vision. A brain MR scan did not show any structural abnormalities, and all standard laboratory tests were in a physiological range. Lumbar CSF pressure in a lateral decubitus position was 32 cm H_2_O. On the basis of all the findings and clinical symptoms the patient was diagnosed with IIH (8). No significant clinical improvement was achieved by corticosteroids, acetazolamide, and topiramate treatment. Therapeutic lumbar puncture also did not relieve the symptoms. However, LP shunt installation instantly improved the child’s vision and relieved the headache. For the following 12 months, the clinical condition was satisfactory, but at the age of 7 years, seldom occasional headaches appeared. At the age of 8 years, a control MR scan showed a tonsillar herniation 25 mm below the level of the foramen magnum, as a complication of LP shunt overdrainage. Headaches increased in frequency, with occasional vomiting, and at the age of 13 years started to interfere with the child’s normal daily activities. Hence, at the age of 14 years suboccipital decompressive craniectomy was performed, but after an initial improvement of symptoms severe headaches appeared accompanied with papilledema. Finally, the LP shunt was replaced with a VP shunt, which resulted in a good clinical outcome and relief of all symptoms.

We performed a volumetric analysis using automated segmentation of the cranial CSF volume on high-resolution T1 slices analyzed by CIVET 1.1.11 software (9) (Montreal Neurological Institute, Montreal, Canada) and manual segmentation of the spinal CSF volume on high-resolution T2 slices analyzed using Analyze 8.1 software (Mayo Clinic, Rochester, MN, USA). The first CSF volumetry was performed at the age of 14 years before suboccipital decompressive craniectomy with the LP shunt installed. The second CSF volumetry was performed at the age of 15 years, 9 months after the suboccipital decompressive craniectomy and replacement of the LP shunt with a VP shunt.

## Results

**Figure 1:**
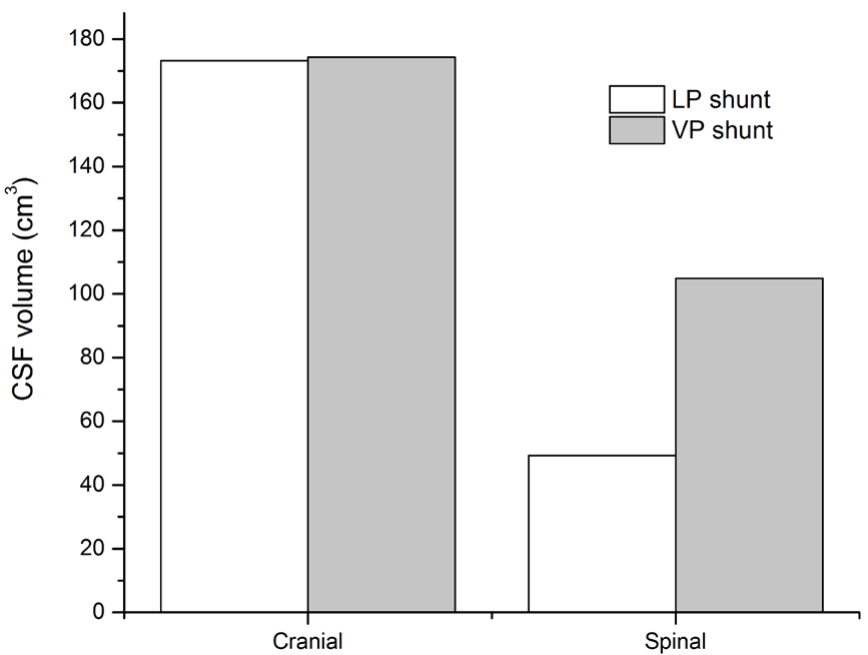
The cranial and spinal cerebrospinal fluid (CSF) volumes in an idiopathic intracranial hypertension (IIH) patient with a lumboperitoneal (LP) shunt (white columns), which was replaced by a ventriculoperitoneal (VP) shunt (gray columns). The cranial CSF volume was the same in both LP (173.2 cm^3^) and VP (174.2 cm^3^) drainage, while the spinal CSF volume was considerably reduced in LP (49.3 cm^3^) compared to VP drainage (104.9 cm^3^).

**Figure 2:**
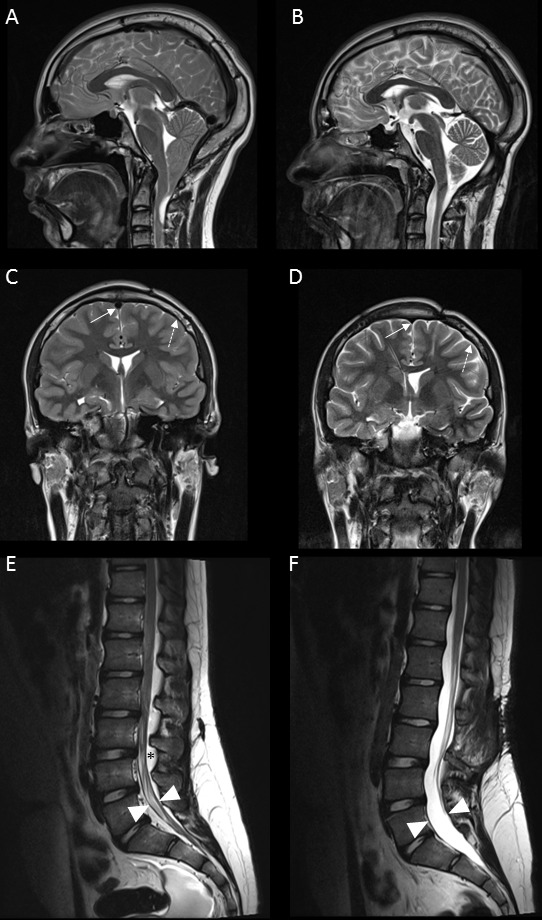
T2 slices of cranial and lumbosacral cerebrospinal fluid (CSF) space in a patient with a lumboperitoneal (LP) shunt (**A,C,E**), which was replaced by a ventriculoperitoneal (VP) shunt **(B,D,F)**. (**A**) Tonsillar herniation through the foramen magnum due to the overdrainage of the LP shunt. (**B**) Appropriate position of the cerebellar tonsils after suboccipital osteoplastic craniotomy and the replacement of the LP shunt with a VP shunt. (**C**) Reduced subarachnoid space (dashed arrow) in the patient with an LP shunt overdrainage accompanied by the normal ventricles size and an increased diameter of the superior sagittal sinus (arrow). (**D**) Reduced size of the right lateral ventricle at the site of the VP shunt insertion with normal findings of other ventricles, the subarachnoid space (dashed arrow), and the superior sagittal sinus (arrow). (**E**) An extremely reduced lumbosacral dural sac (between arrowheads) with an enlarged epidural space (asterisk). (**F**) The lumbosacral dural sac of normal size (between arrowheads) with barely visible epidural tissue.

Volumetric analysis of the CSF space showed that LP shunting induced CSF overdrainage predominantly in the spinal part. The spinal CSF volume was 49.3 cm^3^, which is considerably less than normal (10,11). However, the cranial CSF volume was 173.2 cm^3^, which falls within the normal range (12). Interestingly, after the replacement of the LP shunt with a VP shunt, the cranial CSF volume was not considerably changed and amounted to 174.2 cm^3^, but the spinal CSF volume increased to 104.9 cm^3^ (Figure 1). Thus, the total CSF volume was 56.8 cm^3^ smaller when the LP shunt was in place than when the VP shunt was in place. This difference was a consequence of a reduced spinal CSF volume, while the cranial CSF volume remained the same. Although the intracranial subarachnoid space was somewhat reduced after LP shunt placement (Figure 2C), and the form of the lateral ventricle changed after VP shunt placement at the site of the catheter insertion (Figure 2D), in both conditions the total cranial CSF volume was almost the same.

## Discussion

The presented results suggest that LP shunts reduce the spinal CSF volume, while VP shunts keep the cranial and spinal CSF volume in the physiological range. As after the replacement of the LP with a VP shunt, our patient’s condition improved, it seems that normal cranial and spinal CSF volumes are the preconditions for a good clinical outcome in IIH.

The observed CSF volume changes in our patient can be partially explained by different biophysical characteristics of the cranial and spinal intradural space (13-16) and by different effects of the upright body position on CSF pressures inside the cranial and spinal space. Our previous study (15) has shown that in the horizontal position both cranial and spinal CSF pressures are positive and nearly equal. However, in the upright position the cranial CSF pressure decreases to subatmospheric (negative) values, while the spinal CSF pressure increases and becomes more positive than in the horizontal position (positive value of the spinal CSF pressure corresponds to the distance from the cisterna magna to the lumbar level). So, presumably effective drainage pressures of VP and LP shunts are almost the same in the horizontal position, but after the body position is changed from horizontal to the upright, effective drainage pressure gradient will increase in the case of an LP and decrease in the case of a VP shunt. This is in accordance with the clinical findings in patients with spinal CSF leak, who do not tolerate the upright position well, and in those with cranial CSF leak, who do not tolerate the horizontal position well (17). Also, this could explain frequent complications after LP shunting due to the overdrainage, which leads to the development of significant intracranial hypotension. Intracranial hypotension clinically presents with postural headache in the upright position and as an increased diameter of superficial brain veins or dural sinuses on magnetic resonance (Figure 2C). Severe intracranial hypotension could even lead to tonsillar herniation (Figure 2A). According to our observations, it seems that intracranial hypotension will develop only after a considerable reduction of spinal CSF volume. This is obvious even without detailed and time consuming volumetric analysis because the spinal CSF volume depletion will lead to a decrease in dural sac diameter and an increase in epidural space (Figure 2E). These morphological changes inside the spinal canal are easily detected and evaluated by MRI, which could be used to monitor LP shunt effectiveness and to timely recognize overdrainage complications.

### Conclusion

Our results indicate that VP and LP shunts differently affect the cranial and spinal CSF volume in patients with IIH. This is clearly demonstrated by the overdrainage induced by the LP shunt placement, when only the spinal portion of the CSF was reduced while the cranial portion remained unaffected. Our results emphasize the importance of a total cranial and spinal CSF space evaluation before neurosurgical procedures or during postoperative follow-up, which has been rarely performed so far. Such an evaluation could enable the selection of appropriate therapeutic procedures and early recognition of numerous side effects that occur after the placement of different types of shunts.
